# 1-(Phenyl­sulfon­yl)-1*H*-indole-2-carbaldehyde

**DOI:** 10.1107/S2414314622004011

**Published:** 2022-04-22

**Authors:** Leslie W. Pineda, Natasha Ferllini, Jorge A. Cabezas

**Affiliations:** a Escuela de Química, Universidad de Costa Rica, 11501-2060, San José, Costa Rica; b Centro de Electroquímica y Energía Química (CELEQ), Universidad de Costa Rica, 11501-2060, San José, Costa Rica; University of Aberdeen, Scotland

**Keywords:** crystal structure, heterocyclic system, indole, sulfanilamide

## Abstract

The title compound, C_15_H_11_NO_3_S, was prepared by a facile synthetic approach. The N atom in the pyrrole ring of the indole moiety is pyramidal (bond-angle sum = 350.0°) and the phenyl ring of the phenyl­sulfonyl motif forms a dihedral angle of 76.24 (7)° with the mean plane of the indole ring system. In the crystal, C—H⋯O and C—H⋯π inter­actions link the mol­ecules into a three-dimensional network.

## Abstract 2

In the title indole derivative, which was prepared by a facile synthetic method, the dihedral angle between the aromatic rings is 76.24 (7)°.

## Graphical abstract

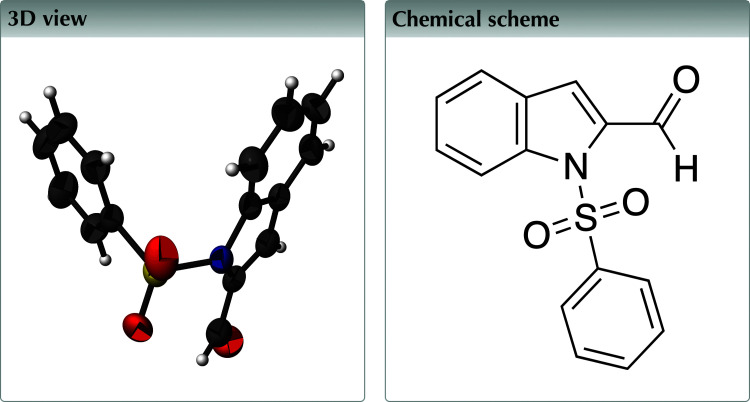

## Structure description

The indole ring framework is a heterocyclic system found in many natural products. Many of these compounds possess biological activity, from neurotransmitter serotonin to vinblastine, an alkaloid clinically used as an anti­cancer agent (Inman & Moody, 2013 ▸). The title compound, **1**, is a useful synthetic inter­mediate, which has been used in the preparation of bouchardatine, a natural occurring alkaloid isolated from the rutaecarpine family (Naik *et al.*, 2013 ▸). It has also been used to synthesize bis­(1*H*-indol-2-yl)methano­nes, potent inhibitors of FLT3 receptor tyrosine kinase (Mahboobi *et al.*, 2006 ▸). Usually, this synthetic inter­mediate is synthesized from indole, which is treated with benzene­sulfonyl chloride under basic conditions, and further formyl­ated at the 2-position by sequential treatment with lithium diisopropyl amide and di­methyl­formamide. As a part of our program of the synthesis of biologically active sulfanilamide derivatives (Cabezas & Arias, 2019 ▸), we report herein a straightforward approach for the synthesis of **1** and its crystal structure.

**Figure 1:**
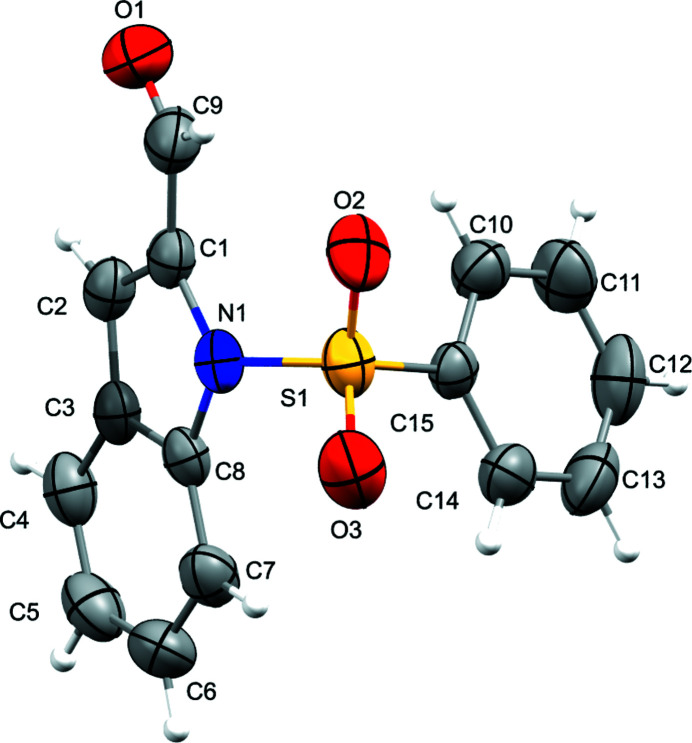
Mol­ecular structure of the title compound with displacement ellipsoids drawn at the 50% probability level.

**Figure 2:**
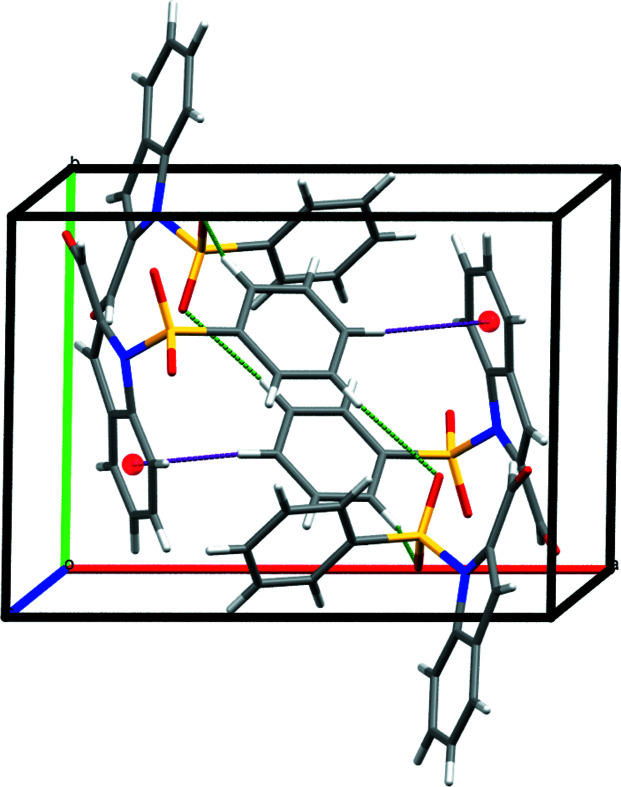
Packing view of the title compound. C—H⋯O and C—H⋯π inter­actions are shown as green and purple dashed lines, respectively.

The crystal structure of **1** has monoclinic symmetry with one mol­ecule in the asymmetric unit: the five-membered pyrrole ring of the indole motif contains a carbaldehyde group and also binds *via* a nitro­gen atom to a phenyl­sulfonyl fragment (Fig. 1 ▸). The bond lengths and angles in **1** do not show any unexpected features (Palani *et al.*, 2006 ▸; Sakthivel *et al.*, 2006 ▸). The bond angles O3—S1—O2 [120.63 (10)°] and N1—S1—C15 [104.80 (8)°] support the distorted tetra­hedral geometry around atom S1. Atom N1 within the pyrrole ring deviates from planar geometry, showing a slight pyramidalization (bond-angle sum = 350.0°). The phenyl ring of the phenyl­sulfonyl motif subtends a dihedral angle of 76.24 (7)° with the mean plane of the indole ring system. There are two short intra­molecular C—H⋯O contacts and the crystal packing features C—H⋯O and C—H⋯π inter­actions (Table 1 ▸, Fig. 2 ▸).

## Synthesis and crystallization

**Figure 3:**
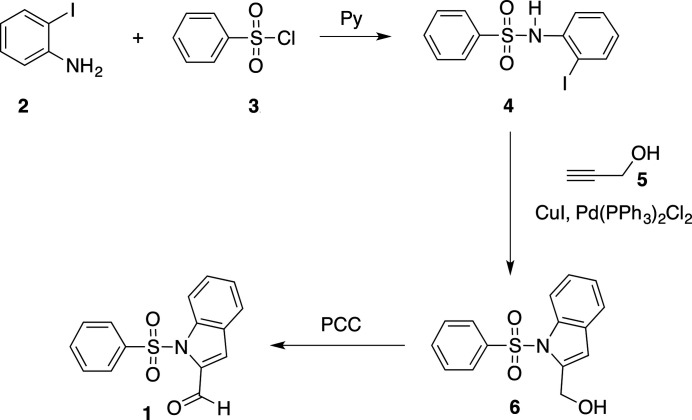
A synthetic scheme for the preparation of the title compound.

The title compound, **1**, was synthesized by the reaction of 2-iodo­aniline, **2**, with benzene­sulfonyl chloride, **3**, in the presence of pyridine to obtain after purification by column chromatography, the iodo­sulfonamide **4**. Treatment of the latter iodide, **4**, with propargyl alcohol, **5**, under Sonogashira’s reaction conditions (Sonogashira *et al.*, 1975 ▸), at room temperature, produced [1-(phenyl­sulfon­yl)-1*H*-indol-2-yl]methanol **6** in a one-pot reaction and with overall yield of 84%. Similar synthetic strategies, using *N*-(2-iodophenyl)methane sulfonamides, required heating at 100–110°C in a sealed tube (Sakamoto *et al.*, 1988 ▸). Oxidation of this alcohol, with pyridinium chlorochromate, provided the target aldehyde in 81% yield (Fig. 3 ▸). The product was recrystallized from ethyl acetate solution at room temperature resulting in light-yellow blocks.

## Refinement

Crystal data, data collection and structure refinement details are summarized in Table 2 ▸.

## Supplementary Material

Crystal structure: contains datablock(s) global, I. DOI: 10.1107/S2414314622004011/hb4405sup1.cif

Structure factors: contains datablock(s) I. DOI: 10.1107/S2414314622004011/hb4405Isup2.hkl

CCDC reference: 2123919

Additional supporting information:  crystallographic information; 3D view; checkCIF report

## Figures and Tables

**Table 1:** Hydrogen-bond geometry (Å, °) *Cg*2 is the centroid of the C3–C8 ring.

*D*—H⋯*A*	*D*—H	H⋯*A*	*D*⋯*A*	*D*—H⋯*A*
C7—H7⋯O3	0.93	2.44	3.014 (3)	120
C9—H9⋯O2	0.93	2.34	2.869 (3)	116
C4—H4⋯O1^i^	0.93	2.51	3.343 (3)	150
C12—H12⋯*Cg*2^ii^	0.93	2.71	3.638 (3)	174

Symmetry codes: (i) 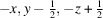 ; (ii) 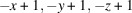 .

**Table 2:** Experimental details

Crystal data	
Chemical formula	C_15_H_11_NO_3_S
*M*_r_	285.31
Crystal system, space group	Monoclinic, *P*2_1_/*c*
Temperature (K)	273
*a*, *b*, *c* (Å)	12.6886 (7), 9.2655 (6), 11.6024 (7)
β (°)	105.374 (2)
*V* (Å^3^)	1315.24 (14)
*Z*	4
Radiation type	Mo *K*α
μ (mm^−1^)	0.25
Crystal size (mm)	0.20 × 0.15 × 0.15

Data collection	
Diffractometer	Bruker D8 Venture
Absorption correction	Multi-scan (*SADABS*; Bruker, 2015 ▸)
*T*_min_, *T*_max_	0.690, 0.746
No. of measured, independent and observed [*I* > 2σ(*I*)] reflections	18696, 3032, 1791
*R*_int_	0.057
(sin θ/λ)_max_ (Å^−1^)	0.651

Refinement	
*R*[*F* ^2^ > 2σ(*F* ^2^)], *wR*(*F* ^2^), *S*	0.048, 0.114, 1.01
No. of reflections	3032
No. of parameters	181
H-atom treatment	H-atom parameters constrained
Δρ_max_, Δρ_min_ (e Å^−3^)	0.24, −0.33

Computer programs: *APEX3* and *SAINT* (Bruker, 2015 ▸), *SHELXT* (Sheldrick, 2015*a* ▸), *SHELXL2014* (Sheldrick, 2015*b* ▸), *Mercury* (Macrae *et al.*, 2020 ▸) and *publCIF* (Westrip, 2010 ▸).

## full crystallographic data

### Crystal data

**Table:**

C_15_H_11_NO_3_S	*F*(000) = 592
*M**_r_* = 285.31	*D*_x_ = 1.441 Mg m^−^^3^
Monoclinic, *P*2_1_/*c*	Mo *K*α radiation, λ = 0.71073 Å
*a* = 12.6886 (7) Å	Cell parameters from 3972 reflections
*b* = 9.2655 (6) Å	θ = 2.8–23.8°
*c* = 11.6024 (7) Å	µ = 0.25 mm^−^^1^
β = 105.374 (2)°	*T* = 273 K
*V* = 1315.24 (14) Å^3^	Block, clear light yellow
*Z* = 4	0.20 × 0.15 × 0.15 mm

### Data collection

**Table:**

Bruker D8 Venture diffractometer	3032 independent reflections
Radiation source: Incoatec Microsource	1791 reflections with *I* > 2σ(*I*)
Mirrors monochromator	*R*_int_ = 0.057
Detector resolution: 10.4167 pixels mm^-1^	θ_max_ = 27.5°, θ_min_ = 2.8°
ω scans	*h* = −16→16
Absorption correction: multi-scan (SADABS; Bruker, 2015)	*k* = −12→12
*T*_min_ = 0.690, *T*_max_ = 0.746	*l* = −15→14
18696 measured reflections	

### Refinement

**Table:**

Refinement on *F*^2^	Primary atom site location: structure-invariant direct methods
Least-squares matrix: full	Secondary atom site location: difference Fourier map
*R*[*F*^2^ > 2σ(*F*^2^)] = 0.048	Hydrogen site location: inferred from neighbouring sites
*wR*(*F*^2^) = 0.114	H-atom parameters constrained
*S* = 1.01	*w* = 1/[σ^2^(*F*_o_^2^) + (0.0529*P*)^2^ + 0.1517*P*] where *P* = (*F*_o_^2^ + 2*F*_c_^2^)/3
3032 reflections	(Δ/σ)_max_ = 0.001
181 parameters	Δρ_max_ = 0.24 e Å^−^^3^
0 restraints	Δρ_min_ = −0.33 e Å^−^^3^

### Special details

**Table:**

Geometry. All esds (except the esd in the dihedral angle between two l.s. planes) are estimated using the full covariance matrix. The cell esds are taken into account individually in the estimation of esds in distances, angles and torsion angles; correlations between esds in cell parameters are only used when they are defined by crystal symmetry. An approximate (isotropic) treatment of cell esds is used for estimating esds involving l.s. planes.
Refinement. All hydrogen atoms were placed geometrically and refined using a riding-model approximation, with C—H = 0.95–1.00 Å and *U*_iso_(H) = 1.2*U*_eq_(C).

### Fractional atomic coordinates and isotropic or equivalent isotropic displacement parameters (Å^2^)

**Table:**

	*x*	*y*	*z*	*U*_iso_*/*U*_eq_	
S1	0.26292 (4)	0.67883 (6)	0.70277 (4)	0.0461 (2)	
O1	0.03455 (14)	0.88036 (19)	0.38393 (17)	0.0771 (5)	
O2	0.23104 (12)	0.82550 (16)	0.70663 (14)	0.0621 (5)	
O3	0.27691 (13)	0.58878 (18)	0.80466 (12)	0.0645 (5)	
N1	0.16726 (12)	0.60075 (18)	0.59334 (13)	0.0407 (4)	
C1	0.11260 (15)	0.6739 (2)	0.48512 (18)	0.0421 (5)	
C2	0.08938 (16)	0.5776 (2)	0.39588 (18)	0.0447 (5)	
H2	0.0523	0.5978	0.317	0.054*	
C3	0.13005 (15)	0.4395 (2)	0.44002 (17)	0.0400 (5)	
C4	0.12749 (18)	0.3046 (3)	0.3870 (2)	0.0537 (6)	
H4	0.0946	0.2926	0.3058	0.064*	
C5	0.17400 (19)	0.1897 (3)	0.4559 (2)	0.0599 (7)	
H5	0.1735	0.0992	0.4211	0.072*	
C6	0.2220 (2)	0.2070 (2)	0.5775 (2)	0.0595 (6)	
H6	0.2527	0.1272	0.6226	0.071*	
C7	0.22548 (18)	0.3386 (2)	0.63281 (19)	0.0519 (6)	
H7	0.2578	0.3491	0.7142	0.062*	
C8	0.17904 (15)	0.4546 (2)	0.56285 (17)	0.0384 (5)	
C9	0.07306 (18)	0.8234 (3)	0.4781 (2)	0.0586 (6)	
H9	0.0781	0.8747	0.5483	0.07*	
C10	0.39626 (19)	0.7687 (2)	0.5702 (2)	0.0523 (6)	
H10	0.3429	0.8375	0.5392	0.063*	
C11	0.4910 (2)	0.7626 (3)	0.5327 (2)	0.0670 (7)	
H11	0.5014	0.8276	0.4756	0.08*	
C12	0.5693 (2)	0.6617 (3)	0.5788 (2)	0.0704 (8)	
H12	0.633	0.6591	0.5537	0.084*	
C13	0.55456 (19)	0.5649 (3)	0.6616 (2)	0.0672 (7)	
H13	0.6078	0.4958	0.692	0.081*	
C14	0.46101 (18)	0.5694 (2)	0.70013 (19)	0.0538 (6)	
H14	0.451	0.5038	0.7569	0.065*	
C15	0.38264 (15)	0.6712 (2)	0.65436 (16)	0.0385 (5)	

### Atomic displacement parameters (Å^2^)

**Table:**

	*U*^11^	*U*^22^	*U*^33^	*U*^12^	*U*^13^	*U*^23^
S1	0.0452 (3)	0.0563 (4)	0.0376 (3)	−0.0103 (3)	0.0123 (2)	−0.0122 (3)
O1	0.0678 (12)	0.0615 (11)	0.0899 (14)	0.0065 (9)	−0.0001 (10)	0.0109 (11)
O2	0.0582 (10)	0.0570 (11)	0.0721 (11)	−0.0043 (8)	0.0187 (8)	−0.0292 (8)
O3	0.0745 (11)	0.0880 (13)	0.0322 (8)	−0.0219 (9)	0.0160 (7)	−0.0020 (8)
N1	0.0362 (9)	0.0476 (11)	0.0390 (10)	−0.0071 (8)	0.0112 (8)	−0.0081 (8)
C1	0.0305 (11)	0.0500 (13)	0.0459 (12)	−0.0037 (10)	0.0106 (9)	−0.0006 (11)
C2	0.0355 (11)	0.0593 (14)	0.0388 (12)	−0.0048 (11)	0.0091 (9)	0.0004 (11)
C3	0.0332 (11)	0.0498 (14)	0.0384 (12)	−0.0092 (10)	0.0119 (9)	−0.0058 (10)
C4	0.0502 (14)	0.0605 (16)	0.0506 (13)	−0.0152 (12)	0.0137 (11)	−0.0164 (13)
C5	0.0657 (16)	0.0443 (14)	0.0737 (18)	−0.0122 (12)	0.0253 (14)	−0.0141 (14)
C6	0.0661 (16)	0.0451 (15)	0.0672 (17)	−0.0050 (12)	0.0175 (13)	0.0078 (13)
C7	0.0575 (15)	0.0532 (15)	0.0433 (13)	−0.0113 (12)	0.0105 (11)	0.0025 (12)
C8	0.0367 (11)	0.0423 (13)	0.0392 (12)	−0.0102 (10)	0.0154 (9)	−0.0040 (10)
C9	0.0426 (14)	0.0569 (16)	0.0715 (17)	−0.0014 (12)	0.0070 (12)	−0.0052 (14)
C10	0.0469 (14)	0.0591 (15)	0.0485 (13)	0.0016 (11)	0.0082 (11)	0.0058 (12)
C11	0.0592 (16)	0.0840 (19)	0.0619 (16)	−0.0138 (15)	0.0235 (13)	0.0115 (14)
C12	0.0386 (14)	0.102 (2)	0.0726 (18)	−0.0104 (15)	0.0182 (13)	−0.0179 (17)
C13	0.0404 (14)	0.0761 (18)	0.0761 (18)	0.0096 (13)	−0.0004 (13)	−0.0081 (15)
C14	0.0515 (14)	0.0565 (15)	0.0476 (13)	−0.0034 (12)	0.0028 (11)	0.0012 (11)
C15	0.0360 (11)	0.0449 (12)	0.0313 (10)	−0.0040 (10)	0.0029 (8)	−0.0027 (10)

### Geometric parameters (Å, º)

**Table:**

S1—O3	1.4191 (15)	C6—C7	1.373 (3)
S1—O2	1.4220 (16)	C6—H6	0.93
S1—N1	1.6708 (16)	C7—C8	1.382 (3)
S1—C15	1.755 (2)	C7—H7	0.93
O1—C9	1.195 (3)	C9—H9	0.93
N1—C8	1.417 (2)	C10—C15	1.375 (3)
N1—C1	1.434 (2)	C10—C11	1.384 (3)
C1—C2	1.339 (3)	C10—H10	0.93
C1—C9	1.468 (3)	C11—C12	1.366 (4)
C2—C3	1.423 (3)	C11—H11	0.93
C2—H2	0.93	C12—C13	1.362 (3)
C3—C4	1.390 (3)	C12—H12	0.93
C3—C8	1.403 (3)	C13—C14	1.376 (3)
C4—C5	1.367 (3)	C13—H13	0.93
C4—H4	0.93	C14—C15	1.371 (3)
C5—C6	1.390 (3)	C14—H14	0.93
C5—H5	0.93		
			
O3—S1—O2	120.63 (10)	C6—C7—C8	117.4 (2)
O3—S1—N1	106.48 (9)	C6—C7—H7	121.3
O2—S1—N1	106.40 (9)	C8—C7—H7	121.3
O3—S1—C15	108.37 (10)	C7—C8—C3	121.63 (19)
O2—S1—C15	109.04 (10)	C7—C8—N1	130.82 (18)
N1—S1—C15	104.80 (8)	C3—C8—N1	107.53 (17)
C8—N1—C1	106.96 (15)	O1—C9—C1	121.2 (2)
C8—N1—S1	119.99 (13)	O1—C9—H9	119.4
C1—N1—S1	123.09 (14)	C1—C9—H9	119.4
C2—C1—N1	108.53 (18)	C15—C10—C11	118.7 (2)
C2—C1—C9	125.7 (2)	C15—C10—H10	120.7
N1—C1—C9	125.00 (19)	C11—C10—H10	120.7
C1—C2—C3	109.67 (18)	C12—C11—C10	120.5 (2)
C1—C2—H2	125.2	C12—C11—H11	119.8
C3—C2—H2	125.2	C10—C11—H11	119.8
C4—C3—C8	119.5 (2)	C13—C12—C11	120.4 (2)
C4—C3—C2	133.3 (2)	C13—C12—H12	119.8
C8—C3—C2	107.27 (18)	C11—C12—H12	119.8
C5—C4—C3	119.0 (2)	C12—C13—C14	120.0 (2)
C5—C4—H4	120.5	C12—C13—H13	120.0
C3—C4—H4	120.5	C14—C13—H13	120.0
C4—C5—C6	120.6 (2)	C15—C14—C13	119.6 (2)
C4—C5—H5	119.7	C15—C14—H14	120.2
C6—C5—H5	119.7	C13—C14—H14	120.2
C7—C6—C5	121.9 (2)	C14—C15—C10	120.8 (2)
C7—C6—H6	119.1	C14—C15—S1	120.28 (17)
C5—C6—H6	119.1	C10—C15—S1	118.89 (16)
			
O3—S1—N1—C8	−53.53 (16)	C4—C3—C8—N1	−178.29 (17)
O2—S1—N1—C8	176.62 (14)	C2—C3—C8—N1	0.5 (2)
C15—S1—N1—C8	61.18 (16)	C1—N1—C8—C7	−179.9 (2)
O3—S1—N1—C1	165.38 (15)	S1—N1—C8—C7	33.4 (3)
O2—S1—N1—C1	35.52 (17)	C1—N1—C8—C3	−1.55 (19)
C15—S1—N1—C1	−79.91 (16)	S1—N1—C8—C3	−148.17 (13)
C8—N1—C1—C2	2.0 (2)	C2—C1—C9—O1	−15.8 (3)
S1—N1—C1—C2	147.38 (14)	N1—C1—C9—O1	175.04 (19)
C8—N1—C1—C9	172.76 (18)	C15—C10—C11—C12	−0.2 (4)
S1—N1—C1—C9	−41.9 (3)	C10—C11—C12—C13	0.7 (4)
N1—C1—C2—C3	−1.7 (2)	C11—C12—C13—C14	−0.7 (4)
C9—C1—C2—C3	−172.36 (19)	C12—C13—C14—C15	0.4 (4)
C1—C2—C3—C4	179.4 (2)	C13—C14—C15—C10	0.1 (3)
C1—C2—C3—C8	0.7 (2)	C13—C14—C15—S1	−179.84 (17)
C8—C3—C4—C5	−0.7 (3)	C11—C10—C15—C14	−0.2 (3)
C2—C3—C4—C5	−179.1 (2)	C11—C10—C15—S1	179.77 (17)
C3—C4—C5—C6	0.7 (3)	O3—S1—C15—C14	11.35 (19)
C4—C5—C6—C7	−0.4 (4)	O2—S1—C15—C14	144.39 (17)
C5—C6—C7—C8	0.0 (3)	N1—S1—C15—C14	−102.03 (17)
C6—C7—C8—C3	0.1 (3)	O3—S1—C15—C10	−168.59 (16)
C6—C7—C8—N1	178.25 (19)	O2—S1—C15—C10	−35.55 (19)
C4—C3—C8—C7	0.3 (3)	N1—S1—C15—C10	78.03 (18)
C2—C3—C8—C7	179.11 (18)		

### Hydrogen-bond geometry (Å, º)

*Cg*2 is the centroid of the C3–C8 ring.

**Table:**

*D*—H···*A*	*D*—H	H···*A*	*D*···*A*	*D*—H···*A*
C7—H7···O3	0.93	2.44	3.014 (3)	120
C9—H9···O2	0.93	2.34	2.869 (3)	116
C4—H4···O1^i^	0.93	2.51	3.343 (3)	150
C12—H12···*Cg*2^ii^	0.93	2.71	3.638 (3)	174

Symmetry codes: (i) −*x*, *y*−1/2, −*z*+1/2; (ii) −*x*+1, −*y*+1, −*z*+1.
